# Psychological distress in cervical cancer screening: results from a German online survey

**DOI:** 10.1007/s00404-020-05661-9

**Published:** 2020-06-27

**Authors:** M. Jentschke, R. Lehmann, N. Drews, A. Hansel, M. Schmitz, P. Hillemanns

**Affiliations:** 1 Department of Gynaecology and Obstetrics, Hannover Medical School, Carl-Neuberg-Str. 1, 30625 Hannover, Germany; 2 DontBePatient Intelligence GmbH, c/o GCI Management, Brienner Str. 7, 80333 Munich, Germany; 3 Oncgnostics GmbH, Winzerlaer Str. 2, 07745 Jena, Germany

**Keywords:** Psychological distress, Cervical cancer screening, Pap smear, HPV test

## Abstract

**Purpose:**

The PODCAD study aimed at assessing the degree of psychological stress that women experience due to notification of an abnormal Papanicolaou (Pap) smear finding or a positive human papillomavirus (HPV) test result.

**Methods:**

We designed a survey to address the question of psychological burden due to abnormal Pap smear results and/or positive HPV tests. In this online campaign approach, we aimed to reach > 2000 women all over Germany irrespective of kind and number of abnormal screening findings. We asked for different kinds of anxiety, distress and uncertainty regarding both, Pap and HPV status.

**Results:**

A total of 3753 women completed the survey at least partially, and almost 2300 fully completed the survey. Of these, more than 50% were affected already since more than 1 year, and almost half of them had experienced at least three Pap smears in follow-up examinations. Almost 70% of the women were afraid of developing cancer. Intriguingly, almost half of the women with abnormal findings were not aware of their stage of the Pap smear. Furthermore, almost 30% of the women displayed signs of a post-traumatic stress disorder.

**Conclusion:**

Abnormal results in cervical cancer screening have an impact on patients’ psychology, irrespective of the knowledge and severity of the findings. Better information concerning risks and benefits of cervical cancer screening and about the meaning of the outcome of its procedures are required to decrease this anxiety.

**Electronic supplementary material:**

The online version of this article (10.1007/s00404-020-05661-9) contains supplementary material, which is available to authorized users.

## Introduction

Cervical cancer is the fourth most common cancer in women worldwide, with more than 500,000 new cases and > 260,000 deaths reported annually [1]. In developed countries, the established screening routines with Pap and/or HPV testing have led to a drastic decrease of incidence and mortality of cervical cancer over the last 5 decades.

On the other hand, both, Pap smear and HPV testing, have relatively low specificity [2–4] leading to a high rate of women with abnormal Pap smear findings or positive HPV results without needing treatment. In Germany, Pap smear is still commonly used and, many women receive notification of abnormal Pap smear results [5]. Cervical cancer is caused by infection with high-risk HPV, and it develops very slowly via so-called cervical intraepithelial lesions. HPV is one of the most common sexually transmitted infections [6]. The fact that vaccination against infection with the most common types of cancer-causing HPV is available has increased the awareness of HPV infection. However, many women are still not aware of the connection between HPV infection and abnormal Pap smear results [7, 8].

Notification of an abnormal Pap smear finding psychologically impacts women, and previous research has aimed at assessing women’s health-related quality of life after such a notification [9–11]. These studies have highlighted that abnormal Pap smear findings evoke negative emotions ranging from anxiety to fear of developing or even having cancer [11–14]. The awareness of the infectious nature of HPV may even increase these emotions [11].

Only a few patients with abnormal Pap smear findings or HPV results are at risk to develop cancer. Most cervical lesions heal spontaneously within 2 years. Furthermore, HPV infections are mostly transient; they clear in about 90% of all cases without even developing a lesion. And in cases of prevalent lesions, neither the Pap smear nor the HPV test is able to distinguish between those lesions which will progress to cancer and those which will heal spontaneously.

As a consequence, Pap smear and/or HPV testing results in a very high number of women having abnormal screening results, and they will have to undergo follow-up testing and/or additional examination such as colposcopy and biopsies of the affected region until the abnormal finding disappears or a decision for surgery (i.e., conization) is made. This sequence of follow-up Pap smears and corresponding examinations such as colposcopies and even biopsies create a burden to women as they will have to stand the ongoing uncertainty whether cancer is already in progress or not.

Several studies are known which collected data from women having abnormal Pap smear findings [15–19] or abnormal findings during screening routine in general [20, 21]. In these studies, study population was rather small, with two exceptions: (1) the TOMBOLA trial comprises 3331 women [18] and (2) Balasubramani and colleagues collected data from 1752 women, but limited to women who underwent colposcopy [22].

We hypothesized that the discussions in recent years regarding HPV infection and its possible prevention through vaccination as well as debates regarding new guidelines for cervical cancer screening would raise the awareness for the psychological burden of women affected. In 2020, Germany will shift from yearly opportunistic cytology screening to co-testing (cytology + HPV test) in three-yearly intervals for women 35 years and older. This will lead to more positive screening results as before because of the lower specificity of HPV testing. Therefore, the problem of psychological distress after positive screening results will be even more evident.

This study was funded by oncgnostics GmbH, a company that aims to commercialize DNA methylation markers. Study design and conception were developed by employees of oncgnostics GmbH.

## Materials and methods

### Survey design

The PODCAD was designed to obtain self-reported patient data on the real-life impact of recurrent abnormal Pap smear findings as well as the current use of the prevention and healthcare system in Germany.

It followed a semi-structured design, combining explorative questions with validated elements.

Participants went through a 37-item survey including the “Impact of Event Scale-Revised”—German Version as well as parts of the Cervical Dysplasia Distress [19, 20, 23] questionnaires, to evaluate the psychological and emotional burden of recurrent abnormal Pap smears.

This semi-structured approach was chosen to allow for the detection of completely independent signals. The complete questionnaire can be found in Supplementary Data 1 and it was assented by the ethics committee of the University Hospital Jena.

### Patient outreach

The PODCAD survey was conducted in two waves during May and June 2018 in Germany and addressed participants purely through an online campaign including channels like display marketing (banner), Search Engine Marketing (i.e., google search) and by link-sharing through the prevention and education campaign F*ck Cancer by “Myriam von M” on facebook (@Myriam.von.M), a community which operates since the beginning of 2014. The PODCAD survey-related online campaign asked, *‘Help us to better understand affected women, a survey on conspicuous PAP/HPV findings*.’

## Results

### Population and Characteristics of participants

A total of 3753 women completed the survey at least partially within 9 weeks. The survey population showed a mean age of 31.8 years and approximately 35.3% of the participants stated to plan for children/additional pregnancies.

To understand the differences between pre-organized (F*ck Cancer Community) and spontaneous participants (from online marketing activities), an interim analysis was conducted comparing the pattern of 902 vs. 89 datasets from the respective groups. This interim analysis did not reveal any significant differences except for a higher proportion (40.6% vs. 34.8%) of women with active family planning attitude in the Online Marketing cohort. We interpreted this to be the main driver for the actual web research activity. In conclusion, both cohorts can be analyzed together.

The results presented below are based on approximately 2300 complete surveys which fulfilled quality criteria and represented the target population, women with at least one abnormal Pap smear. However, almost half (46.6%) of the participating women indicated that they had 3–5 (32.1%) or even more (14.5%) conspicuous Pap smears (Table 1). More than half of the women (53.1%) have already been affected for more than 1 year and claimed to be burdened by the conspicuous Pap smear results.

**Table 1:** Number of suspicious cytology test results

Suspicious cytology test results	
1×	32.7%
2×	17.3%
3–5 times	32.1%
more than 5 times	14.5%
I’m not quite sure	3.4%

**Fig. 1:**
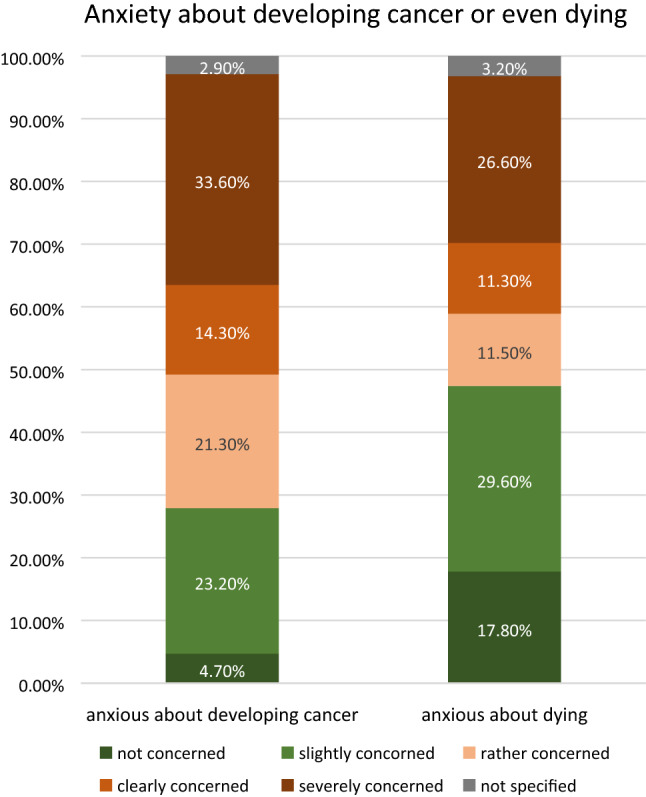
Anxiety about developing cancer or even dying

Although only 40.9% seemed to know their exact Pap smear findings (Table 2), more than 2 out of 3 women (69.3%) stated to be afraid of developing or being diagnosed with cervical cancer, and almost half (49.4%) expressed that they were even afraid to die of cancer (Fig. 1). Finally, it is worth to note that the knowledge about the Pap smear results seems to be largely independent of the level of education/degree.

**Table 2:** Distribution of different cytology findings among women who knew the actual cytology result (40.9% of all participants)

Knowing the Pap result?	
Yes, I know my actual Pap finding	40.9%
Pap I	21.0%
Pap II	20.2%
Pap III	13.3%
Pap IIID	27.6%
Pap IVa	8.7%
Pap IVb	2.0%
Pap V	0.9%
I‘m not quite sure	6.6%

### Questionnaire outcome

#### Concerns about abnormal Pap smear findings/positive HPV test results

**Fig. 2:**
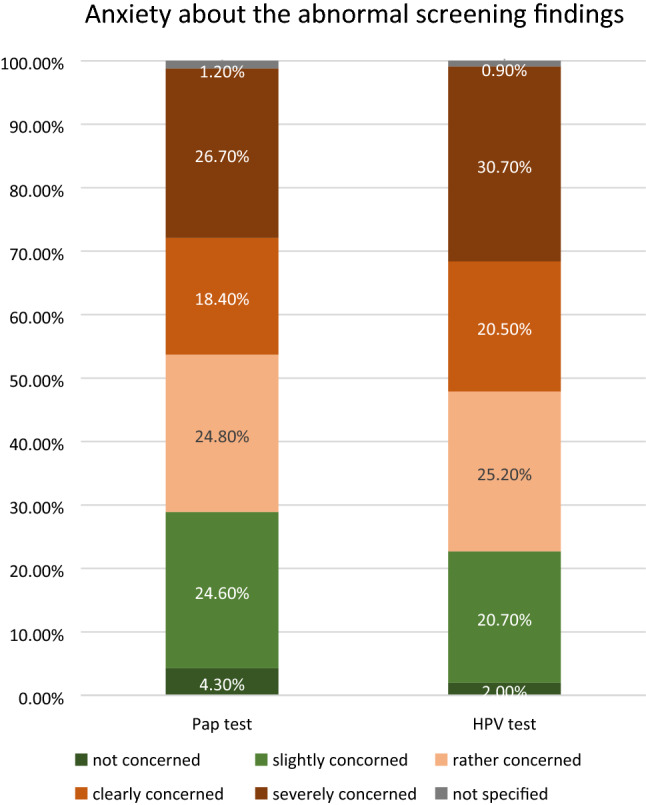
Anxiety about the abnormal screening findings. Women with abnormal Pap smear findings or positive HPV test results were asked to judge, on a scale from 0 to 5 (1 = not concerned; 2 = slightly concerned; 3 = rather concerned; 4 = clearly concerned; 5 = severely concerned), how much they were concerned about these findings

In consideration of the ongoing and unclear situation of conspicuous results, more than two-third of the women with abnormal Pap smear findings (69.9%) and more than ¾ of the participating women with positive HPV test result (76.4%) expressed to be at least “rather concerned” (Scores 3, 4 and 5 on a 5-point scale—Fig. 2) about the findings, respectively. 26.7% (PAP) and 30.7% (HPV) reported to be even “severely concerned” (Score 5 on a 5-point scale—Fig. 2).

#### Influence on family planning/future pregnancies

**Fig. 3:**
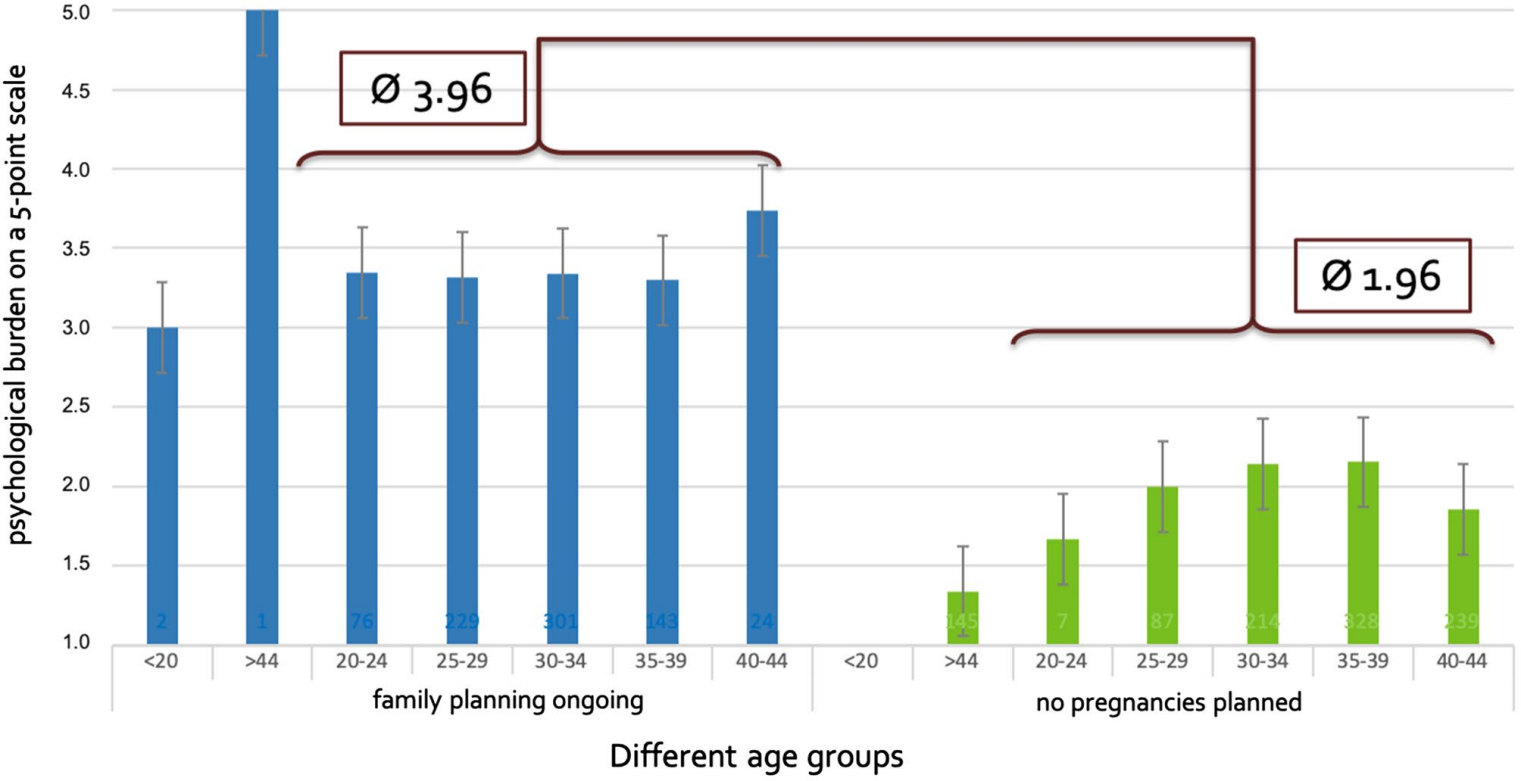
Distress level among women with childbearing preferences is higher than among other women. Average level of concerns of women, with or without childbearing preferences, in different age groups (< 20 years; 20–24 years; 25–29 years; 30–34 years; 35–40 years; 40–44 years; > 44 years)

In addition, women who claimed to still be planning pregnancies show a significantly higher burden (average score 3.96 vs. 1.96—Fig. 3), irrespective of their age. Nearly half of the participants (48.1%) stated that the risk of conizations as well as the risk of preterm birth is important to them and “clearly” to “severely” impacting their life (Scores 4 and 5 on a 5-point scale—Fig. 4, supplemental data). In one of the four participants (25%) this has already influenced the family planning.

In conclusion, more than half of the participants (53.1%) have to deal/cope with the described situation and conspicuous Pap/HPV findings for more than 1 year.

#### Post-traumatic stress

For 28% of the women who completed the “Impact of Event Scale-Revised” scale (n = 794) that allows for assessing their post-traumatic stress disorder, results above the cut-off value in the sense of a post-traumatic stress disorder were achieved (Fig. 5, supplemental data).

#### Seeking clarification

Women suffering from positive Pap- or HPV test results are clearly seeking clarification. If there would be a test which could clarify their situation, 98% would want to receive such additional testing. Of these, more than half (56.6%) are willing to pay themselves for a clarification test, 35.9% are possibly willing to pay. Women still in family planning phase would pay more than women not planning further pregnancies (Fig. 6, supplemental data).

## Discussion

In this study, we investigated the experience of women with abnormal Pap smear findings and positive HPV test results using a semi-structured design. With more than 3700 participants, of whom almost 2300 filled the questionnaire completely, this study, to our knowledge, is one of the largest within this field. Among women with at least one abnormal Pap smear finding the degree of completeness of the questionnaire increased with the number of Pap smears the women had experienced. Overall, almost half of those women had experienced more than three, and each seventh woman even more than five abnormal Pap smears. This means that watchful waiting strategies that last for longer times are followed frequently.

Intriguingly, many women (40%) did not know their exact Pap smear finding, indicating that more exact information is required when the woman gets informed by the practitioner/gynecologist. Often, women only know that their Pap is abnormal but they cannot distinguish between small abnormalities and high-grade dysplasia. Therefore, it seems to be necessary that the meaning of the abnormal Pap smear finding is well explained. In this context, it may be problematic that this information is usually given by the affected women via letter sometimes together with an appointment for the next control smear. The same holds true for the diagnosis of a high-risk HPV infection. If HPV alone is tested and diagnosed, the average chance to clear the infection is around 90%. So, also in cases of HPV infections, women need clear information what this infection means for them. These findings are in line with a very detailed review from Frederiksen and colleagues [24]. They summarized the data from 16 different studies measuring psychological outcome in women with a histological diagnosis or treatment of cervical intraepithelial neoplasia or even cervical cancer. One of the key findings was that the psychological outcome of women with cervical intraepithelial neoplasia was similar to those of women with abnormal cytology but not necessarily needing treatment. They interpreted this finding as a consequence of the uncertainty of the women about their true disease status. Would the women be better informed, we would not expect that such a high proportion of them, almost 70% in our survey outcome, would be afraid to develop cancer. Frederiksen et al. also saw that women considered a cervical intraepithelial neoplasia diagnosis to be as bad as that of cervical cancer. Even more intriguing is the fact that 50% of the women with abnormal Pap smear finding or positive HPV test result are afraid to die of cancer. With better information, they would be aware of the fact that “only” every third woman diagnosed with invasive cervical cancer dies of the disease, and that, if the disease is diagnosed early, the chance for a complete cure is very high.

Women who receive information about a suspicious screening result often try and look for further information in the internet. There they are often misleaded by false information and reports from women affected with cervical cancer.

In Germany, an organized screening program started in 2020 and the responsible working groups have considered the psychological burden which can occur after screening results partially in their procedures. The information letter, which is sent to every woman 20 years and older every 5 years, gives information about the meaning of the finding “cervical intraepithelial neoplasia 1, 2, 3” and “suspicious Pap” and the women are also informed about the fact that if attending the screening program regularly, the chance for a false-positive results once a year is very high [25].

Most cervical lesions, depending upon their severity, heal within 1–2 years, a well-known fact that justifies the watchful waiting strategies followed by the gynecologists which then leads to the current situation reflected in the results of our study. Besides better information, improved diagnostic testing options which allow a better assessment of abnormal Pap smears and positive HPV tests would be very helpful. These would, on the one hand, reduce anxiety if they had very good specificity. Frederiksen and colleagues drew the same conclusion, that avoiding false-positive results would substantially decrease screening-induced anxiety [24]. And it would allow for timely treatment of relevant disease, which then results in a very high chance for successful treatment.

Our questionnaire comprised, besides explorative questions, also validated elements. One of these, the Cervical Dysplasia Distress Questionnaire [19] aims at measuring the women’s perception of diagnostic procedures, their distress and sexual concerns concerning a precancerous genital lesion [20]. The other element, the “Impact of Event Scale-Revised”, allows to estimate if some of the women experience signs of post-traumatic stress disorder which is known to significantly reduce quality of life and influence morbidity and mortality [26, 27]. This was the case for almost 30% of those almost 800 women who filled this part of the questionnaire completely and properly. This is in line with former findings [22, 28–30], where the severity of screening-induced anxiety was also comparable to those after traumatic events.

Women do not seem to be aware of the fact that most HPV infections are transient and that even most high-grade cervical lesions do not progress to cancer. This is reflected by the observation in our study that more than half of the women with a positive HPV test result are “clearly concerned” about this finding. The observation that women are even more concerned about positive HPV results compared to suspicious Pap test findings is in contrast to a study from Kitchener and colleagues [31], in which they could not see an impact on distress by receiving a positive HPV test result in addition to a positive Pap test compared to a positive Pap test alone. However, the structure of their questionnaire was a bit different and, e.g., HPV test alone was not addressed.

Limitations of the PODCAD survey include the participant selection bias and whether this is a representative sample of conspicuous Pap/HPV patients, as the survey was only accessible to those with internet access. Also, because this survey invited all individuals with conspicuous Pap/HPV findings to take part, those with the higher burden are more likely to have responded compared with those whose overall scoring was lower. Additionally, a bias may have been introduced when recalling past information such as Pap smear scorings received and impact on daily activities. This is reflected by the fact that 40% of the participants did not know their exact Pap smear result.

However, it should be noted that the completion rate of the survey was above the mean compared to surveys of similar length (n = 19), thus operating in the range of chronic pain disorders. Particularly high completion rates of surveys are observed associated with pain disorders, rare diseases and situations of high suffering. The PODCAD survey is the first of its kind to investigate the psychological distress during recurrent diagnostics cycles from patients with conspicuous Pap/HPV findings and has highlighted important findings in relation to the unmet clinical needs of the participants. Better awareness of the burden of disease and available diagnostic or treatment options are needed and may help to improve real-life patient care.

## Conclusion

From the PODCAD survey, we conclude that in Germany, the psychological distress during recurrent diagnostics cycles within conspicuous Pap/HPV findings is unmet or uncontrolled in a significant proportion of the patients. The PODCAD survey exposed a large proportion of conspicuous Pap/HPV “sufferers” who have apparently unmet needs for a clear prognosis or diagnosis. The burden was often felt to be higher in the context of family planning. The survey concluded with the question, if the participants would be willing to perform an additional test to receive a clear “cancer: yes/no” result and 98% would want to undergo additional testing. Of these, 92.5% would even pay ≥ 50€ for such a clarification test. A positive family planning history leads to even significantly higher willingness for additional testing.

## Electronic supplementary material

Below is the link to the electronic supplementary material.

Women’s fear concerning risks of conizations, such as preterm birth, after an abnormal Pap smear finding.

Indications for Post-traumatic stress disorder among women with abnormal Pap smear findings or positive HPV tests, as judged from their answers in the “Impact of Event Scale-Revised” part of the questionnaire.

Prices women are willing to pay for a clarification test.

## Data Availability

All data are made available upon request
