# Supplementary figures and images for: Psychological distress in cervical cancer screening: results from a German online survey
Source: Arch Gynecol Obstet. 2020 Jun 27;302(3):699–705. doi: 10.1007/s00404-020-05661-9 (PMC7447652; doi:10.1007/s00404-020-05661-9)

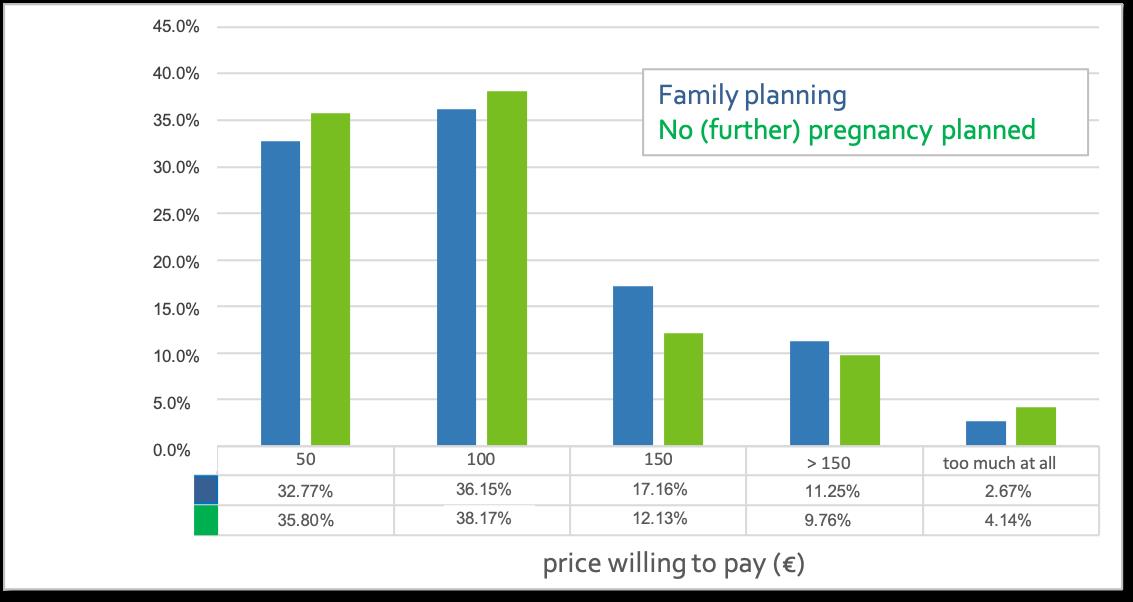

Supplement: Supplementary file 3 — Prices women are willing to pay for a clarification test. [file 404_2020_5661_MOESM3_ESM.jpg]
