# Supplementary material for: Psychological distress in cervical cancer screening: results from a German online survey
Source: Arch Gynecol Obstet. 2020 Jun 27;302(3):699–705. doi: 10.1007/s00404-020-05661-9 (PMC7447652; doi:10.1007/s00404-020-05661-9)
Supplement: Supplementary file 2 [file 404_2020_5661_MOESM1_ESM.docx]

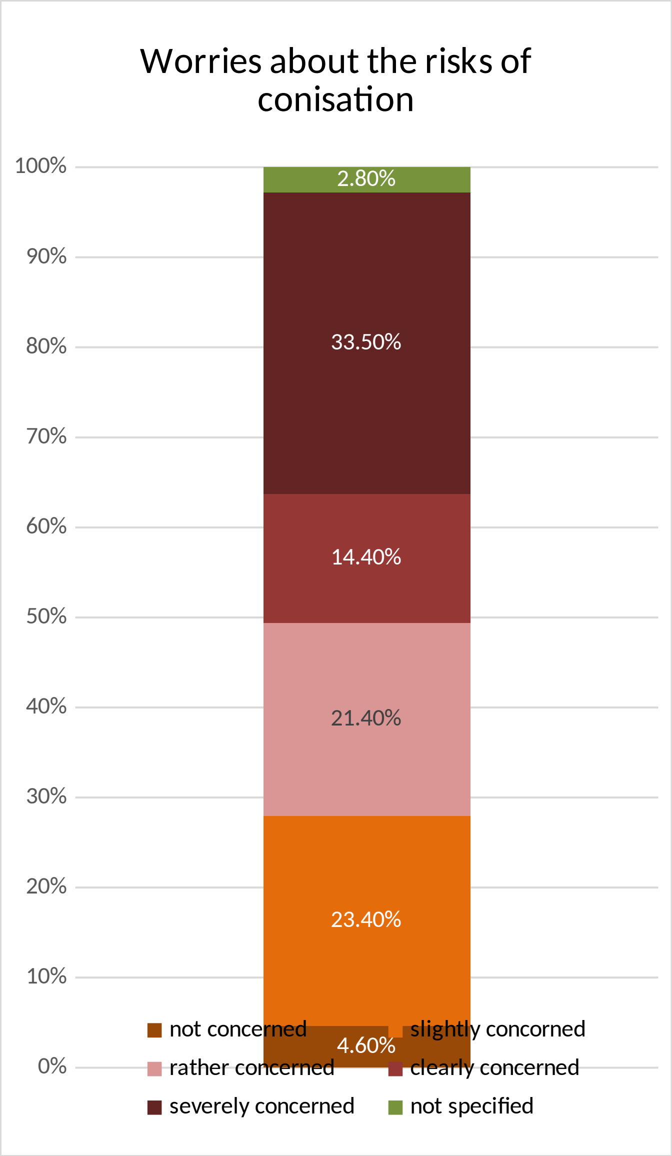
Worries about the risks of conisation
100%
90%
80%
70%
60%
50%
40%
30%
20%
10%
0%
2.80%
33.50%
14.40%
21.40%
23.40%
4.60%
not concerned
slightly concorned
rather concerned
clearly concerned
severely concerned
not specified
